# Metagenomic next-generation sequencing contributes to the diagnosis of mixed pulmonary infection: a case report

**DOI:** 10.1186/s12941-022-00545-z

**Published:** 2022-11-24

**Authors:** Ziqian Qin, Yiwu Zou, Zehe Huang, Ning Yu, Zhenfeng Deng, Zhencheng Chen, Yuanli Wang

**Affiliations:** 1 Clinical Genome Center, Guangxi Kingmed Diagnostics, Nanning, 530007 Guangxi China; 2 The First People’s Hospital of Qinzhou, The Tenth Affiliated Hospital of Guangxi Medical University, Qinzhou, 535000 Guangxi China; 3 Guilin University of Electronic Technology, Guilin, 541004 Guangxi China

**Keywords:** Metagenomic next-generation sequencing, Pulmonary cryptococcosis, Bacterial pneumonia, Mixed pulmonary infection, Diagnosis

## Abstract

**Background:**

Pulmonary cryptococcosis (PC) and mixed pulmonary infection are difficult to be diagnosed due to the non-specificity and their overlapping clinical manifestations. In terms of the clinical diagnosis of PC and mixed pulmonary infection, conventional tests have limitations such as a long detection period, a limited range of pathogens, and low sensitivity. Metagenomics next-generation sequencing (mNGS) is a nascent and powerful method that can detect pathogens without culture, to diagnose known and unexplained infections in reduced time.

**Case presentation:**

A 43-year-old female was admitted to the hospital after suffering from a cough for one month. At the time of admission, a contrast-enhanced chest CT revealed multiple nodules and plaques in her right lung, as well as the formation of cavities. The blood routine assays showed evidently increased white blood cell count (mainly neutrophils), CRP, and ESR, which suggested she was in the infection phase. The serum CrAg-LFA test showed a positive result. Initially, she was diagnosed with an unexplained pulmonary infection. Bronchoalveolar lavage fluid (BALF) samples were collected for microbial culture, immunological tests and the mNGS. Microbial culture and immunological tests were all negative, while mNGS detected *Corynebacterium striatum*, *Pseudomonas aeruginosa*, *Streptococcus pneumoniae*, and *Cryptococcus neoformans*. The diagnosis was revised to PC and bacterial pneumonia. Lung infection lesions were healed after she received targeted anti-infection therapy with mezlocillin and fluconazole. In a follow-up after 2 months, the patient’s symptoms vanished.

**Conclusions:**

Here, we demonstrated that mNGS was capable of accurately distinguishing *Cryptococcus* from *M. tuberculosis* in pulmonary infection, and notably mNGS was capable of swiftly and precisely detecting pathogens in mixed bacterial and fungal pulmonary infection. Furthermore, the results of mNGS also have the potential to adjust anti-infective therapies.

**Supplementary Information:**

The online version contains supplementary material available at 10.1186/s12941-022-00545-z.

## Background

Pulmonary cryptococcosis (PC) is a lung infection caused by inhaling *Cryptococcus neoformans* or *Cryptococcus gattii* in the environment, which are two major cryptococcal pathogens in humans and animals; these two pathogens belong to the *Cryptococcus neoformans* species complex. When Cryptococcus spores are inhaled, they frequently enter the human lower respiratory tract. It is most common in immunocompromised patients. However, PC becomes one of the emerging diseases in immunocompetent patients [1], and epidemiological surveys in recent years have shown that the proportion of asymptomatic infections in immunocompetent PC patients is higher than that in immunocompromised patients (40.8% and 30.2%, respectively) [2]. For immunocompetent patients, their clinical symptoms are different from immunodeficiency patients [3]. PC can be confirmed by some conventional tests, e.g. taking specimens by lung puncture biopsy for culture, direct microscopy examination, and serum cryptococcal capsular polysaccharide antigen lateral flow assay (CrAg-LFA) test. CrAg-LFA detection is currently one of the most important methods for early diagnosis of *cryptococcosis* because it’s fast and low-cost. But the clinical manifestations of PC are non-specific [4], with cough, expectoration and fever being the most common symptoms. Moreover, Chest computed tomography (CT) of PC patients may show multiple nodular masses and cavities in some lesions [5], and some imaging of PC is similar to that of pulmonary tuberculosis, which may cause misdiagnosis. Since the clinical images of PC vary with the immune status of patients, and clinical symptoms are nonspecific and diverse [6], PC is easily misdiagnosed as other diseases, such as pulmonary tuberculosis, pneumonia and lung cancer. What is worse, patients have died in some cases due to the severe exacerbation of the disease because PC is misdiagnosed as pulmonary tuberculosis [7, 8].

Recently the incident rate of mixed pulmonary infection has increased, with mixed bacterial and fungal infections being common [9]. Mixed pulmonary infection is more common in the elderly or patients with compromised immunity and immunodeficiency. Different pathogens can cause mixed pneumonia at the same time, resulting in overlapping clinical manifestations that make it difficult to identify all pathogens in the early stage of diagnosis [10]. In addition, the long-term empirical treatment with broad-spectrum antibiotics may increase drug resistance in some pathogens. Conventional tests for infectious pathogens, such as microbial culture, microscopy methods, antigen detection techniques, and serologic tests, have deficiencies like long average detection turn-around time (TAT), low sensitivity and low positive rate. These shortcomings increase the risk that a mixed pulmonary infection is missed or misdiagnosed, hindering patients from receiving effective care in the timeliest manner possible.

Metagenomic next-generation sequencing (mNGS), an emerging pathogen detection method in recent years, can directly detect and identify pathogens by sequencing without culturing or classifying. Here, we retrospectively analyzed a female diagnosed with bacterial pneumonia complicated by PC and showed how mNGS contributes to the identification of infectious pathogens during diagnostic procedures.

### Case presentation

**Fig. 1:**
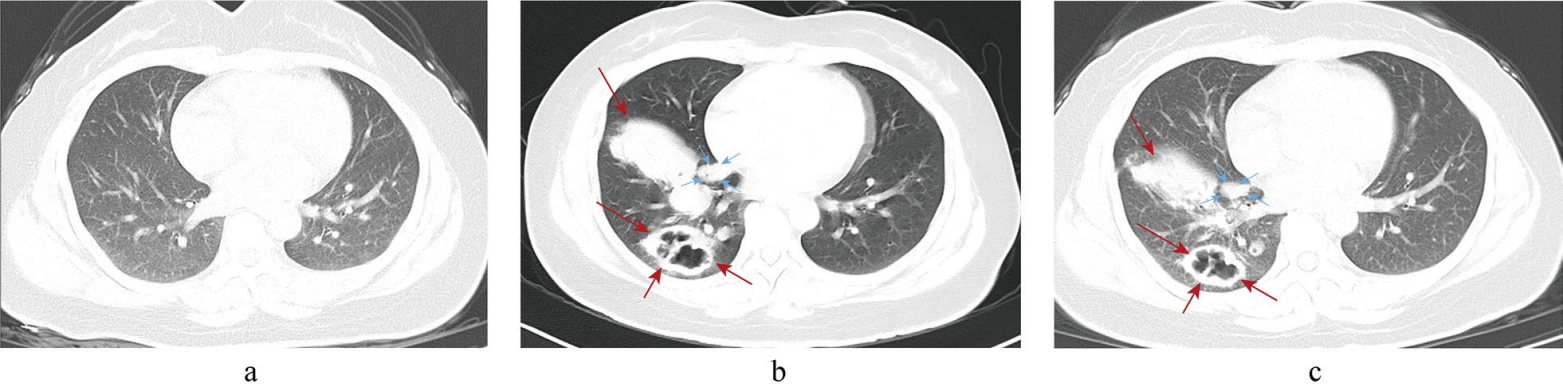
Comparisons of chest CT without infection one year before admission (**a**), contrast-enhanced chest CT on the second day of admission (**b**) and chest CT on the 18th day of admission (**c**). **a** The CT imaging showed no no abnormality. **b** The CT imaging showed plaque shadowing and obvious cavity formation in the right lung, as well as multiple nodules. **c** The lesions had obvious absorption

A 43-year-old female was admitted to the Department of Respiratory and Critical Care Medicine, the First People’s Hospital of Qinzhou, because she had been coughing for over a month. One day before hospital admission, her chest CT at another local hospital showed space-occupying lesions in the right lower lobe, along with cavities and multiple nodules. Her preliminary diagnosis was a pulmonary infection. She was admitted to the hospital for further diagnosis and treatment. About 10 months ago, she underwent right plantar cyst resection at the Department of Joint and Sports Medicine of the hospital; the surgery was successful and she made a smooth recovery. She denied a history of cigarette-smoking, tuberculosis, intrafamilial infectious diseases, and a family history of genetic diseases. Physical examinations at the time of admission were normal, with the following parameters: body temperature 36.2 °C, breathing rate 20 times/min, heart rate 112 times/min, and blood pressure 116/83 mmHg. Her right lower lung breath sounds were slightly coarse, while her right upper lung and left lung breath sounds were clear, and there were no obvious rales or pleural friction rub in both lungs. Auxiliary examinations were as followed: WBC 12.32 × 10^9^/L, absolute neutrophil count = 8.48 × 10^9^/L, CRP = 16.04 mg/mL, ESR = 72.00 mm/h; serum CrAg-LFA test was positive; the *M. tuberculosis* antibody TB-Dot test was weakly positive; no abnormalities were found in G and GM test, liver and kidney function, blood gas, tumor markers test, and squamous cell carcinoma antigen examinations. Her contrast-enhanced chest CT image revealed multiple nodules and plaques in her right lung, as well as the formation of cavities, which is considered the characteristic of tuberculosis, but the fungal infection could not be ruled out (Fig. 1b). She was diagnosed with pulmonary infection due to the nature of the space-occupying lesions and cavities in the right lower lobe, and moxifloxacin (0.4 g, qd, by intravenous drip) was used for empirical anti-infective therapy.

**Fig. 2:**
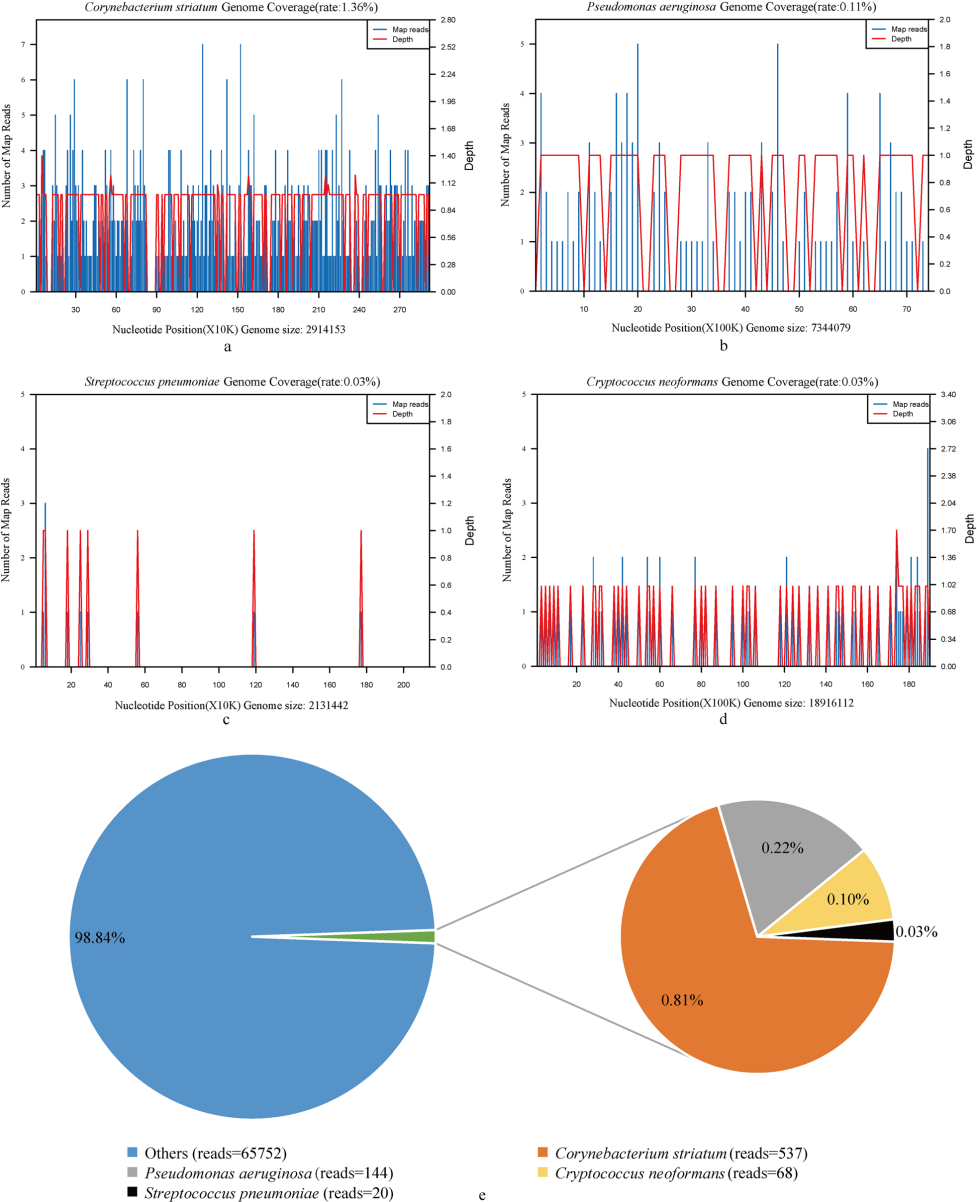
The mNGS result of the patient's BALF sample. Coverage graph of pathogen: *Corynebacterium striatum* (**a**), *Pseudomonas aeruginosa* (**b**), *Streptococcus pneumoniae* (**c**), and *Cryptococcus neoformans* (**d**). The abscissa represents the genome position, the left ordinate is the number of reads of the pathogen aligned on the genome location (Number of Map Reads shown in blue), and the right ordinate is the average sequencing depth at the corresponding position (Depth, the red line in the figures). The proportion of pathogen sequences in microbe detected in her BALF sample (e): The orange section is *Corynebacterium striatum* (0.81%), the gray section is *Pseudomonas aeruginosa* (0.22%)*,* the gold section is *Cryptococcus neoformans* (0.10%), the black section is *Streptococcus pneumoniae* (0.03%), and the blue section is other microorganisms

Chronologically, on the3rd day of admission, her tuberculin skin test (PPD) was negative. On the 7th day, her body temperature was 36.5 °C. Her cough did not improve significantly, and she experienced occasional chest pain. No abnormality was found in bronchoscopy, and BALF samples were collected for microbial culture, G and GM test, *M. tuberculosis* DNA test, and fungal and *Cryptococcus* smear, all of which proved negative. In the meantime, a BALF specimen was directly sent to Clinical Genome Center, Guangxi Kingmed Diagnostics, for mNGS in accordance with the previous references [11, 12]. On the 9th day, the mNGS result (see Additional file 1 for the methods of mNGS) reported a total of 66521 single-end reads in the pathogen genomic DNA, of which 537 reads were *Corynebacterium striatum*, with a coverage of 1.37% (Fig. 2a). 144 reads were *Pseudomonas aeruginosa*, with a coverage of 0.11% (Fig. 2b). 20 reads were *Streptococcus pneumoniae* with a coverage of 0.03% (Fig. 2c), and 68 reads were *Cryptococcus neoformans* with a coverage of 0.03% (Fig. 2d). The species’ relative abundance values of *Corynebacterium striatum*, *Pseudomonas aeruginosa*, *Streptococcus pneumoniae* and *Cryptococcus neoformans* were 15.23%, 4.08%, 0.57% and 60.71%, respectively. There were no reads that were matched to *M. tuberculosis*. With the lung images and laboratory examination results in hand, the clinician considered a high possibility of bacterial and cryptococcal infection. Therefore, the diagnosis was changed to PC and bacterial pneumonia, and the anti-infection therapy was switched to mezlocillin (4 g, q8h, by intravenous drip) for antibacterial treatment and fluconazole sodium chloride injection (400 mg, qd, by intravenous drip) for antifungal treatment. Over the next 7 days, the patient gradually improved after anti-infective treatment, and because the treatment is effective, the current anti-infective regimen was maintained. On the 18th day, the re-examination CT showed that her right lung infection lesions were significantly reduced (Fig. 1c). On the 19th day, the woman was discharged from the hospital, and in a follow-up call two months later she self-reported that her symptoms had mostly subsided.

## Discussion and conclusions

Here, we described a case that the results of the conventional methods of the patient were inaccurate, self-contradictory and confusing: the serum CrAg-LFA test was positive, and *M. tuberculosis* antibody TB-Dot was weakly positive, whereas the microbial culture, smear, G test, *M. tuberculosis* DNA test (BALF) and PPD test were all negative. Tuberculosis infection cannot be excluded due to inconsistency in *M. tuberculosis* related tests and the chest CT imaging similarities to imaging symptoms of *M.tuberculosis* infection. To acquire more accurate information, taking a BALF sample for mNGS was used to aid in the exclusion of *M. tuberculosis* infection; this is mainly because in conjunction with the serum CrAg result, G test was negative and imaging cannot help to exclude the possibility of *M. tuberculosis* infection. The sequences of *Cryptococcus neoformans* were detected by mNGS, which was consistent with the CrAg-LFA result (a gold standard [4]). Eventually, based on all these results, PC was diagnosed. There have been many reports of PC being misdiagnosed as pulmonary tuberculosis [3, 6]. One reason is that patients’ early symptoms and imaging manifestations have no specificity [13]. The continuous development and popularization of mNGS in recent years has compensated for the shortcomings of traditional methods, especially for infections with non-specific imaging or clinical manifestations, because mNGS’s high sensitivity improves the pathogens’ detection rate. Furthermore, in one study, Ning Zhu et al. showed only a 27.3% sensitivity of BALF culture for PC diagnosis in their case [14]. Therefore, relying on etiological examination for PC diagnosis seems to be a less efficient method.

Due to the overlapping of imaging symptoms and the non-specificity of clinical manifestations, the diagnosis of mixed pulmonary infection is difficult. There are inadequacies of conventional detection methods that prevent timely treatment of patients with mixed pulmonary infection and have negative impacts. First of all, conventional methods—such as microbial culture of respiratory samples, microbial smears, G and GM test—have low sensitivity and cover a narrow range of pathogens, so diagnosis, based on the clinicians’ assumptions and inferences, must rely heavily on clinicians’ extensive experience. In our case, the bacterial culture was negative, while mNGS accurately detected the mixed infection of three kinds of bacteria, which provides a broader diagnosis and treatment option for the clinic.Furthermore, the long TAT of conventional methods is one of the reasons for the procrastination in the identification of pathogens in mixed pulmonary infection. According to research [15], the average TAT of conventional tests is over 3 days, while the TAT of mNGS only takes 2 days. In our case, the mNGS result was report within 40 h. Long TAT of traditional detection methods and a complex diagnosis and treatment route, cause the diagnosis of mixed infections and uncommon pathogens to take a long time, which is one of the reasons for poor prognosis [16]. What is more, long-term empirical treatment with broad-spectrum antibiotics has led to the emergence of drug resistance in some pathogens, and the drug resistance itself is increasing. Also, the early use of broad-spectrum antibiotics is one of the reasons for the low positive rate of conventional detection methods [17]. mNGS is unbiased and has broad pathogens coverage, which greatly offsets the shortcomings of conventional tests for the detection of mixed infection. It can quickly identify multiple pathogens of mixed pulmonary infection without bias as well as assumption and minimize missed diagnosis, lessening the abuse of antibiotics as a consequence of inexperience and the long TAT of conventional methods. Jiahui et al. [17] analyzed 55 cases of mixed pulmonary infection and found that the sensitivity of mNGS in diagnosing mixed pulmonary infection was about 7 times higher than that of conventional tests (97.2–13.9%, respectively). Besides, the results of mNGS can assist clinicians to adjust anti-infective therapy. Tianjun et al. [18] discovered that that 60.6% of the patients showed apparent improvement after following mNGS results, wheras only 37.9% of the patients diagnosed by conventional test improved. In our case, the mNGS result was a mixed infection of bacteria and *Cryptococcus neoformans,* while the bacterial culture and smear of BALF samples were both negative. Clinicians modified the anti-infectious treatment plan in time, which diminished the time of using broad-spectrum antibiotics.

It is noteworthy that mNGS can quickly and unbiasedly detect and identify pathogens in mixed-infected patients without culture, and it has more outstanding sensitivity than traditional tests [19], particularly in detecting fungi, viruses, and anaerobic bacteria. The high sensitivity of mNGS makes it easier for positive pathogens to be detected in samples even if these pathogens are with a low load. Because of the advantages of high sensitivity, broad coverage of pathogens, and short TAT [7], mNGS can help clinicians to quickly and accurately identify unknown etiologies [20]. The high pathogen coverage and unbiasedness of mNGS play a great role in the differential diagnosis of pathogens causing lung infections, including mixed pulmonary infections, rare pathogen infections, and the differential diagnosis of some bacteria and fungi. There have been numerous reports of misdiagnosed and missed-diagnosed patients whose diagnoses were modified after mNGS tests [21]. However, it is undeniable that mNGS still has certain limitations. In our case, one of the reasons for the low proportion of pathogen reads detected is that most of the detected microorganisms were reagent engineering microorganisms and colonization microorganisms (*Malassezia*, *Sphingomonas*, and *Acinetobacter* in our case). This drawback can be overcome by optimizing the bioinformatics analysis process and using negative control. In addition, other issues involve the interference of human source sequences with pathogen detection and low detection efficiency for pathogens with cell wall protection. The optimization can include removing human source sequences, and optimizing protocols to enable rupturing cell walls more easily for releasing pathogens’ nucleic acids.

In conclusion, we present a case where the diagnosis was modified from an unexplained pulmonary infection to PC and bacterial pneumonia after mNGS test, demonstrating the potential role of mNGS in distinguishing PC from pulmonary tuberculosis when conventional test results are inconsistent and precisely detecting pathogens in mixed pulmonary infection. Compared with some traditional methods, mNGS can detect pathogens more swiftly and accurately with higher sensitivity, which can assist clinicians to identify pathogens with similar imaging manifestations, and greatly reduce the time of diagnosis. Additionally, the patient in our case recovered after anti-infection therapy was adjusted to a suitable one, which also suggests that mNGS results have the potential ability to adjust treatment regimens. It is very meaningful because it may reduce the abuse of antibiotics, and providing clear ideas for precise anti-infectious treatment plans. Although there are still some limitations of mNGS at this stage, they are expected to be gradually improved with the constant update and optimization of the experimental protocols and bioinformatics analyses, and mNGS is suitable to be used for assisting clinical diagnosis of mixed pulmonary infection.

## Supplementary Information

**Additional file1.** Methods of mNGS.

## Data Availability

The data supporting the conclusions of this article are included within the article.

## Abbreviations

PC: Pulmonary cryptococcosis
CT: Computed tomography
mNGS: Next-generation sequencing
CrAg-LFA: Cryptococcal capsular polysaccharide antigen lateral flow assay
BALF: Bronchoalveolar lavage fluid
TAT: Turn-around time
