# Supplementary material for: Metagenomic next-generation sequencing contributes to the diagnosis of mixed pulmonary infection: a case report
Source: Ann Clin Microbiol Antimicrob. 2022 Nov 24;21:52. doi: 10.1186/s12941-022-00545-z (PMC9701064; doi:10.1186/s12941-022-00545-z)
Supplement: Supplementary file 1 — Additional file1. Methods of mNGS. [file 12941_2022_545_MOESM1_ESM.docx]

**Additional file 1. Methods of mNGS**

The methods of mNGS in Guangxi KingMed Diagnostics were the same as that described in a previously published mNGS article which was conducted in Guangzhou KingMed Diagnostics[1]

1. Sample Processing

Bronchoalveolar lavage fluid (BALF) sample was collected and transported with dry ice.

2. DNA extraction and library preparation

In this study, nucleic acid (DNA) extraction and library preparation were performed on samples through the lab's self-built process. After the BALF sample was enriched, DNA extraction was performed following JIANSHI BIOTECH Universal DNA/RNA extraction protocol (Catalog No. TR202JY-50). Library prepared following KingCreate Biotechnology Pathogenic Microorganism Metagenomic DNA Detection Protocol (Reversible End Termination Sequencing, Catalog No. KS619-DNAmN48)[2, 3].

3. Sequencing and Quality Control

Both nucleic acid extraction and library preparation were conducted in parallel with quality control samples. Single-end 75bp sequencing was carried out using Illumina Nextseq 550 System with 75 cycles Reagent Kit.

4. Bioinformatic Analysis

After filtering the low-quality sequencing data by fastp v0.20.0[4] and removing the sequences mapped to the human reference genome using bwa v0.7.10-r789[5], the remaining data were aligned to the microbial genome database. The alignment of the remaining microbial data was carried out using bwa v0.7.10-r789[5] and Sequence-Based Ultra-Rapid Pathogen Identification (SURPI v1.0.18) pipeline (UCSF), which is a previously published research pipeline for pathogen identification[6, 7]. A microorganisms database “MetagenomicX” was built for clinical use to align the sequencing data, which contains 36497 microorganisms’ genomes covering most of microorganisms that have been sequenced.

References:

1. Shi Y, Chen J, Shi X, Hu J, Li H, Li X, et al. A case of chlamydia psittaci caused severe pneumonia and meningitis diagnosed by metagenome next-generation sequencing and clinical analysis: a case report and literature review. BMC infectious diseases. 2021;21:1–8.

2. Miao Q, Ma Y, Wang Q, Pan J, Zhang Y, Jin W, et al. Microbiological diagnostic performance of metagenomic next-generation sequencing when applied to clinical practice. Clinical Infectious Diseases. 2018;67 suppl_2:S231–40.

3. Editorial Board of Chinese Journal of Infectious Diseases. Clinical practice expert consensus for the application of metagenomic next generation sequencing. Chin J Infect. 2020;38:681–9.

4. Chen S, Zhou Y, Chen Y, Gu J. fastp: an ultra-fast all-in-one FASTQ preprocessor. Bioinformatics. 2018;34:i884–90.

5. Li H, Durbin R. Fast and accurate short read alignment with Burrows–Wheeler transform. bioinformatics. 2009;25:1754–60.

6. Miller S, Naccache SN, Samayoa E, Messacar K, Arevalo S, Federman S, et al. Laboratory validation of a clinical metagenomic sequencing assay for pathogen detection in cerebrospinal fluid. Genome research. 2019;29:831–42.

7. Naccache SN, Federman S, Veeraraghavan N, Zaharia M, Lee D, Samayoa E, et al. A cloud-compatible bioinformatics pipeline for ultrarapid pathogen identification from next-generation sequencing of clinical samples. Genome research. 2014;24:1180–92.
